# The Role of Sex and Gender in Dermatology - From Pathogenesis to Clinical Implications

**DOI:** 10.1177/12034754231177582

**Published:** 2023-07-04

**Authors:** François Lagacé, Kathleen D’Aguanno, Connor Prosty, Alexandra Laverde-Saad, Leila Cattelan, Lydia Ouchene, Sarah Oliel, Genevieve Genest, Philip Doiron, Vincent Richer, Abdulhadi Jfri, Elizabeth O’Brien, Philippe Lefrançois, Mathieu Powell, Linda Moreau, Ivan V. Litvinov, Anastasiya Muntyanu, Elena Netchiporouk

**Affiliations:** 1 5620 Division of Dermatology, Faculty of Medicine, McGill University, Montréal, Québec, Canada; 2 5620 Faculty of Medicine, McGill University, Montréal, Québec, Canada; 3 5620 Division of Allergy and Immunology, Faculty of Medicine, McGill University, Montréal, Québec, Canada; 4 12366 Division of Dermatology, Faculty of Medicine, University of Toronto, Toronto, Ontario, Canada; 5 233826 Department of Dermatology and Skin Science, University of British Columbia, Vancouver, BC, Canada; 6 1811 Department of Dermatology, Brigham and Women’s Hospital/Dana-Farber Cancer Institute, Harvard Medical School, Boston, MA, USA

**Keywords:** sex, gender, dermatology, incidence, skin condition

## Abstract

**Background:**

Sex and gender have increasingly been recognized as significant risk factors for many diseases, including dermatological conditions. Historically, sex and gender have often been grouped together as a single risk factor in the scientific literature. However, both may have a distinct impact on disease incidence, prevalence, clinical presentation, severity, therapeutic response, and associated psychological distress.

**Objectives and project description:**

The mechanisms that underlie differences in skin diseases between males, females, men, and women remain largely unknown. The specific objectives of this review paper are:

1. To highlight the biological differences between males and females (sex), as well as the sociocultural differences between men and women (gender) and how they impact the integumentary system.
2. To perform a literature review to identify important sex- and gender-related epidemiological and clinical differences for various skin conditions belonging to a range of disease categories and to discuss possible biological and sociocultural factors that could explain the observed differences.
3. To discuss dermatological skin conditions and gender-affirming treatments within the transgender community, a population of individuals who have a gender identity which is different than the gender identity they were assigned at birth.

**Future impact:**

With the rising number of individuals that identify as non-binary or transgender within our increasingly diverse communities, it is imperative to recognize gender identity, gender, and sex as distinct entities. By doing so, clinicians will be able to better risk-stratify their patients and select treatments that are most aligned with their values. To our knowledge, very few studies have separated sex and gender as two distinct risk factors within the dermatology literature. Our article also has the potential to help guide future prevention strategies that are patient-tailored rather than using a universal approach.

## Introduction

Sex and gender have been increasingly recognized as significant risk factors for many diseases, including dermatological conditions. Sex represents the biological differences between males and females and includes anatomical, physiological, endocrine, genetic, and morphological traits.^1^ On the other hand, gender (short for gender norms, gender roles, and gender expression) encompasses the sociocultural, psychological, and behavioral differences between men and women, such as self-representation, roles, habits, and activities that are perceived as either masculine or feminine according to society, culture, and/or religion.^1-3^ Historically, sex and gender have often been grouped together as a single risk factor in the scientific literature. However, this creates bias in research data and clinical care as both have a distinct impact on disease incidence, prevalence, clinical presentation, severity, therapeutic response, and psychological distress.^2^

Through gene expression, epigenetic modifications, hormone levels, immune system functioning, and variations in skin anatomy, sex can exert effects on cutaneous disease onset, etiology, and progression in a multitude of ways. Likewise, through variations in lifestyle, diet, stress, environmental/occupational exposures, and socio-cultural behaviors, gender can exert its effects on disease risk, clinical presentation, healthcare access, and treatment compliance. Thus, sex and gender can interplay to mediate substantial effects on patients’ disease at all stages. Sex and Gender Medicine must therefore be recognized as a critical step in improving patient-centered care,^2^ for individuals identifying as transgender, gender fluid, and/or non-binary as well as cisgender individuals.

The specific objectives of this paper are to 1) review the biological differences relevant to dermatology between males and females (sex), as well as the sociocultural differences between men and women (gender); 2) describe and discuss important sex- and gender-related epidemiological and clinical disparities for various skin conditions; and 3) discuss skin diseases in the transgender community.

## Terminology

For the purpose of this article, the terms *male* and *female* refer to individuals with XY and XX sex chromosomes, respectively, and the terms *men* and *women* refer to one’s gender identity, which is defined as the internal experience and knowledge of oneself as a man, woman, non-binary, or as another form of gender identification.^4,5^ The terms men and women are also used to discuss gender, which includes sociocultural norms and learnt gender behaviors.^4,5^

## Part 1 – Underlying Concepts

### Sex: Biological Differences

**Figure 1:**
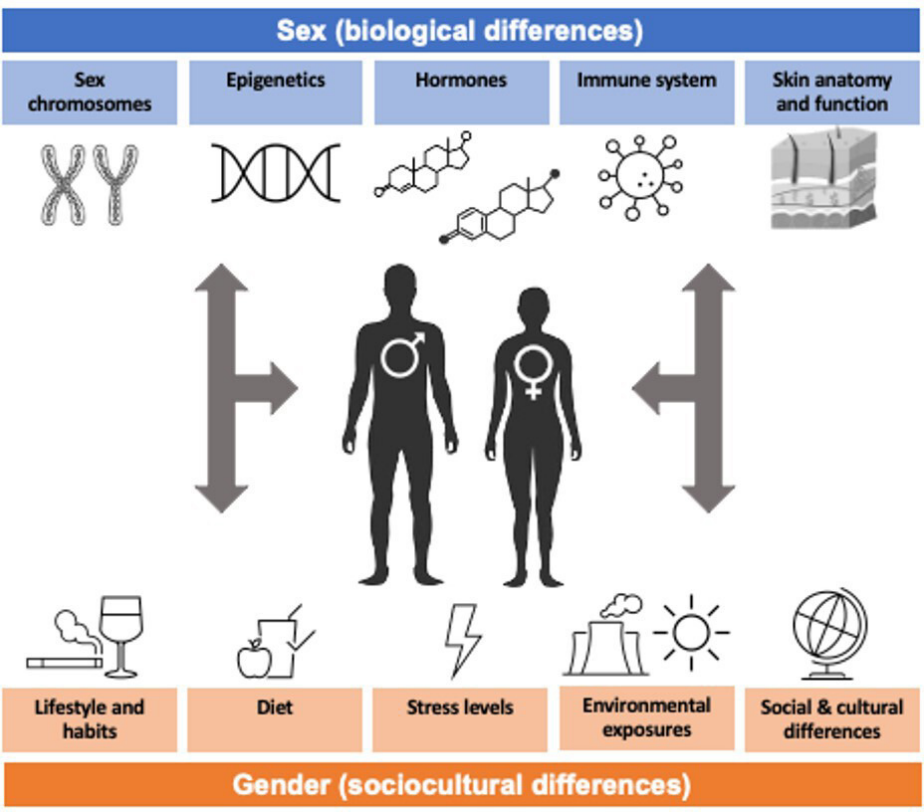
Summary of biological (sex) and sociocultural (gender) factors that differ between men/males andwomen/females

Multiple biological and physiological differences between males and females account for the observed sexual dimorphism in skin diseases. Genetics along with epigenomic modifiers are crucial for XX *vs*. XY genotype, influencing the hormone milieu and immune function. In Part 1, we will review basic concepts in genetics, hormones, immune system, skin anatomy and function that contribute to these observed differences (Figure 1).

#### Genetics and epigenetics

The XY or XX chromosomes are functional in all cell types.^6^ The presence of Y chromosome determines male gonadal differentiation and hormone expression, whereas its absence underlies female gonadal differentiation and hormonal milieu. The X chromosome contains many additional autosomal-like genes that contribute to phenotypic differences notably through modulation of immunity and epigenetics.^2,6-8^

The gene expression of sex chromosomes is also modulated by lyonization, imprinting and additional epigenetic modifications explaining heritability concepts beyond the genotype.^7^ Lyonization (*i.e.,* X chromosome inactivation in females) leads to mosaicism and increased genetic heterogeneity in females.^2,6,7,9^ This mosaicism becomes visible to dermatologists in female patients heterozygous for an X-linked disease, where the disease phenotype is milder in females compared to males and/or follows a mosaic or patchy distribution (e.g., Incontinentia Pigmenti and Goltz syndromes) ^10,11^. Genomic imprinting further influences expression or inactivation of genes depending on which parent it was inherited from.^7,12^ Histone modification, noncoding RNAs, and DNA methylation are additional mechanisms behind silencing or activation of sex and autosomal genes’ transcription.^13^ While epigenetic modifications are complex and far from being fully understood, they are influenced by both biological factors (*e.g.,* immunity and hormones) and external influences, which include sociocultural and gender-related factors, whereby sex and gender concepts closely interact with each other.^13^

#### Hormones

The effect of sex hormones in general health and disease is discussed elsewhere.^13^ Briefly, their influence starts in the embryo affecting morphogenesis and continues throughout life affecting multiple genes, receptors, and the neurochemical axis. The importance of sex hormones during embryogenesis and its clinical implications are summarized in the Supplemental Material.^13^ In dermatology, sex hormones affect both skin homeostasis and immune responses, as receptors for estrogen, progesterone, androgens, and prolactin are generally present on numerous cells of the epidermis, dermis, and innate and adaptive immune systems.^2,6,7,13,14^ In particular, estrogens are believed to be activators of the immune response, whereas androgens and progesterone are suppressors. This leads to an overall stronger immune response in females compared to males, as well as a predilection for autoimmunity in females.^2^

Estrogens prolong the anagen phase of hair growth through increased cell proliferation, increase skin thickness and collagen content, maintain skin moisture, increase sebum production, promote wound healing, and have antioxidant properties.^14-17^ Progesterone exhibits mainly androgen-like effects on the pilosebaceous unit explaining acne flare-ups perimenstrual or with external progesterone contraceptives.^18-20^ Androgens are crucial in the development and maintenance of the pilosebaceous unit, regulation of hair growth, secretion and production of sebum, wound healing, and skin barrier formation.^14,16,21^ Local or systemic androgen overproduction may clinically translate into seborrhea, acne/hidradenitis suppurativa, hirsutism, and androgenetic alopecia.^6-8,21,22^ Prolactin promotes keratinocyte proliferation and regulates keratin expression and sebum production. It has inhibitory effects on hair growth, and plays a role in osmoregulation and thermoregulation.^23^ Hormonally influenced life events such as puberty, menopause/andropause, and pregnancy or external hormonal replacement therapies influence skin homeostasis and alter the risk of various skin diseases. Further information concerning the effects of estrogen, progesterone and androgens on the immune system is summarized in Supplemental Tables 1-3. Hormonal variation during life events (e.g., puberty, pregnancy, post-partum period, menopause, andropause) and their impact on skin homeostasis is summarized in Supplemental Table 4.

#### Immune System

In general, the innate, cellular, and humoral immune responses are all heightened in females as discussed above.^6,24^ Many pro-inflammatory genes are present on X chromosomes, and therefore have a higher level of expression in females. Antigen presentation and phagocytic activity of innate immune cells are more efficient in females than in males.^8^ There are no differences in total lymphocyte counts between sexes; however, the proportion of *T* cells and the CD4:CD8 ratio is lower in males.^6,7^ Females generate stronger humoral and cellular immune responses to antigens.^7^ Notably, females have higher IgM levels at all ages and produce higher levels of circulating antibodies in response to an antigen or a vaccine.^6,7,25^ T-cell differential is also different across sexes. Females have an increased tendency to develop a Th1 response and generate more inflammatory cytokines.^26^ On the other hand, males produce a stronger Th17 response (which may correlate with more severe psoriasis seen in males, see Part 2) and, over the years, immunosenescence with Th2 skewing is more pronounced in males than in females.^6,27,28^ Females’ heightened immunity contributes to their lower risk of infection/cancer, which is hypothesized to provide a survival advantage during the reproductive years but predisposes them to autoimmunity (discussed in Part 2). It has been hypothesized that inactivation of genes involved in immunity by lyonization contributes to this predisposition.^29,30^

#### Skin Anatomy and Function

The epidermis has the same thickness, and the stratum corneum has the same mass, thickness, hydration, and adhesion in both sexes.^8,14^ Males carry more organisms on the skin, and there is conflicting evidence with regards to sex differences in total epidermal water loss and skin pH.^8,14,26^ Dermal collagen is denser and as a result the dermis is thicker in males; however, whether this translates into clinical importance is unknown.^8,14^ Males and females both demonstrate gradual dermal thinning starting in the second and fifth decade, respectively.^8^ The subcutaneous fat is thicker in females.^8^

Males have higher sebum content, especially on the face, as well as overall higher sweat rates than females, which predisposes males to diseases involving sebaceous glands, including seborrheic dermatitis and acne.^8,14,31,32^ In terms of cutaneous microvasculature, females have decreased basal blood flow and demonstrate vasodilation in response to local heat at lower temperatures than males do, possibly leading to a lower sweat response in females.^14^

#### Pharmacokinetics

There exists a multitude of ways in which sex may affect an individual’s response to therapeutics.^33^ Drug absorption can vary by sex through differences in gastric pH (more acidic in males), varying levels of expression of gastric and intestinal enzymes, and shorter gastrointestinal transit time in males (44.8 hr vs. 91.7 hr) ^33^. Drug distribution is influenced by a variety of parameters including plasma volume, body mass index, average organ blood flow, total body water, free plasma protein concentration, and cardiac output which are generally higher in males, as well as body fat, which is higher in females. The larger volume of adipose tissue results in an increased distribution of lipophilic drugs in females.^34^ There also exists established sex-related differences in drug metabolism as females have higher activity of certain CYP450 enzymes (CYP3A4 and 2D6). Drug elimination can vary by sex as renal blood flow and pulmonary function are increased in males.^33^ These parameters are also influenced by age and hormonal life events (*e.g.,* pregnancy and menopause) ^33,35^. For topical medications, owing to increased dermal thickness, absorption is decreased in males whereas it is increased in pregnant females due to increased dermal hydration and blood flow.^33^

### Gender: Sociocultural Differences

At the population level, there are many occupational, environmental, recreational, social, and cultural differences between men and women. Not only are the types and frequency of exposures often different between men and women, but the host’s responses to these exposures also differ and may have an impact on disease severity.^7^ In this section, we will review lifestyle and habits, diet, environmental and occupational exposures, stress levels, and cultural differences between men and women.

#### Lifestyle and Habits

In 2018, Statistics Canada reported gender differences in several health parameters. Overall, men have a higher frequency of daily cigarette smoking (12.3% vs. 9.4%), e-cigarette use (8.8% vs. 3.3%), marijuana use (9.0% vs. 5.9%), heavy alcohol consumption (23.5% vs. 14.8%), and are less likely to have a regular health care provider (81.6% vs. 88.9%). However, men are more likely to exercise at least 150 minutes per week than women (59.1% vs. 50.2%).^36-38^ These habits have many effects on the integumentary system, as described in Supplemental Table 5.

#### Diet

Diet and nutritional patterns differ between men and women. Women tend to avoid foods with high fat contents, are more likely to restrict salt and consume more fruits and vegetables. They are more likely to eat when experiencing stress or in group settings, and experience higher levels of frustration based on their diet-related choices.^1,39,40^ In contrast, men tend to prefer fattier foods and meats, as well as foods with a stronger taste. Typically, they eat more snacks while watching television, consume more dietary supplements, and eat “fast food” more commonly.^1,41^ Overall, men have higher rates of obesity (28.0% vs. 25.6%) which contributes in part to skin disease risk such as psoriasis, intertrigo, etc. (discussed in Part 2).^36-38^

#### Stress Levels

According to Statistics Canada, 19.1% of men (95% CI 18.3-20.0) and 22.9% of women (95% CI 22.0-23.7%) report that on most days their stress level is ‘quite a bit’ or ‘extreme’.^36^ In addition, the proportion of individuals that self-reported ‘fair’ or ‘poor’ perceived mental health is 6.7% (95% CI 6.1-7.3%) and 8.6% (95% CI 8.1-9.3%) in men and women, respectively.^36^ Although multiple biological factors, such as regulation of the hypothalamus-pituitary-adrenal axis, have been reported to contribute to the difference in stress responses observed in men and in women, studies have shown that gender-related factors also play an important if not predominant role.^42-44^ Stress has a significant impact on skin homeostasis, a process that is mediated by the ‘brain-skin’/neurocutaneous axis.^45^ Indeed, high stress levels lead to altered immune function. Acute stress responses lead to immune activation in the skin with increased migration of Langerhans cells to lymph nodes. Clinical translation of this includes exacerbation of chronic spontaneous urticaria (CSU) or *de novo* onset of alopecia areata (AA) following stress.^46^ In contrast, chronic stress leads to immunosuppression, impaired wound healing, altered barrier function, and lower resistance to infection.^45^ This axis can also contribute to inflammation by causing the release of neuropeptides, neurotrophins, cytokines/lymphokines, and other mediators to the skin.^47^ The impact of stress on the skin is also illustrated by psychocutaneous and neurocutaneous diseases (discussed in Part 2).

#### Environmental Exposures

Men and women are exposed to different environmental, recreational, and occupational exposures. In terms of occupational exposures and tasks, overall, men report higher exposures to dust, chemical substances, loud noises, welding fumes, herbicides, wood dust, solvents, medical radiation, vibrating tools, and physically demanding work.^48,49^ While rare, this may translate into development of some gender-specific occupational dermatoses such as Erasmus syndrome (systemic sclerosis (SSc) seen in men exposed to silica at work). On the other hand, women are more frequently exposed to disinfectants, hair dyes, textile dust, and are more likely to perform repetitive tasks.^48^ One European study found that, at home, women use more household cleaning products, decorative cosmetic products, hair dyes, and more personal care products.^50^ This may lead to different presentations of contact dermatitis among men and women for instance Riehl melanosis being observed more commonly in women (Part 2).^51^ The impact of a given exposure on one’s health is determined not only by the substance itself, but also by the body’s absorption and excretion of the substance, which can vary between sexes as explained in Part 1.^3^ Men also report working more night shifts and irregular hours than women.^48^ Though the pathophysiology has yet to be fully elucidated, shift work has been linked to an increased risk of developing metabolic diseases, including metabolic syndrome and type 2 diabetes.^52,53^ It has been hypothesized that disruption of circadian rhythm promotes stress response and consequently a pro-inflammatory state.^54^ Men spend more time outdoors than women.^8^ Indeed, compared to women, men report higher UV exposure, which has immunosuppressive properties, in addition to decreased sun protective behaviors predisposing men to higher rates of skin cancer.^6^

#### Social and Cultural Differences

At the population level, men and women exhibit significant differences in terms of social practices. Certain theories suggest that gender differences are the result of sociocultural expectations and gender roles, gender socialization, and power differentials within a societal structure.^39,55^ In addition, multiple sociocultural factors are believed to play a role in the observed habits and life circumstances that differ between men and women as social groups, including social organization, dietary habits, roles in the households, culture, economic conditions, and education.^39^ These factors have an impact on men and women’s health, on both a population and individual level, and can vary from one culture to another. For example, some cultures may perceive driving a car or being employed as more masculine behaviors and staying at home or caring for the family as more feminine behaviors.^39,44^ Another example illustrating the sociocultural impact on gender-related behaviors are gender-specific hair and nail styles, which vary between cultures.^8^ These various sociocultural differences lead to different exposures between men and women, which could have an impact on the health of individuals.^3^

## Part 2 - Sex and Gender Differences in Dermatological Diseases

**Figure 2:**
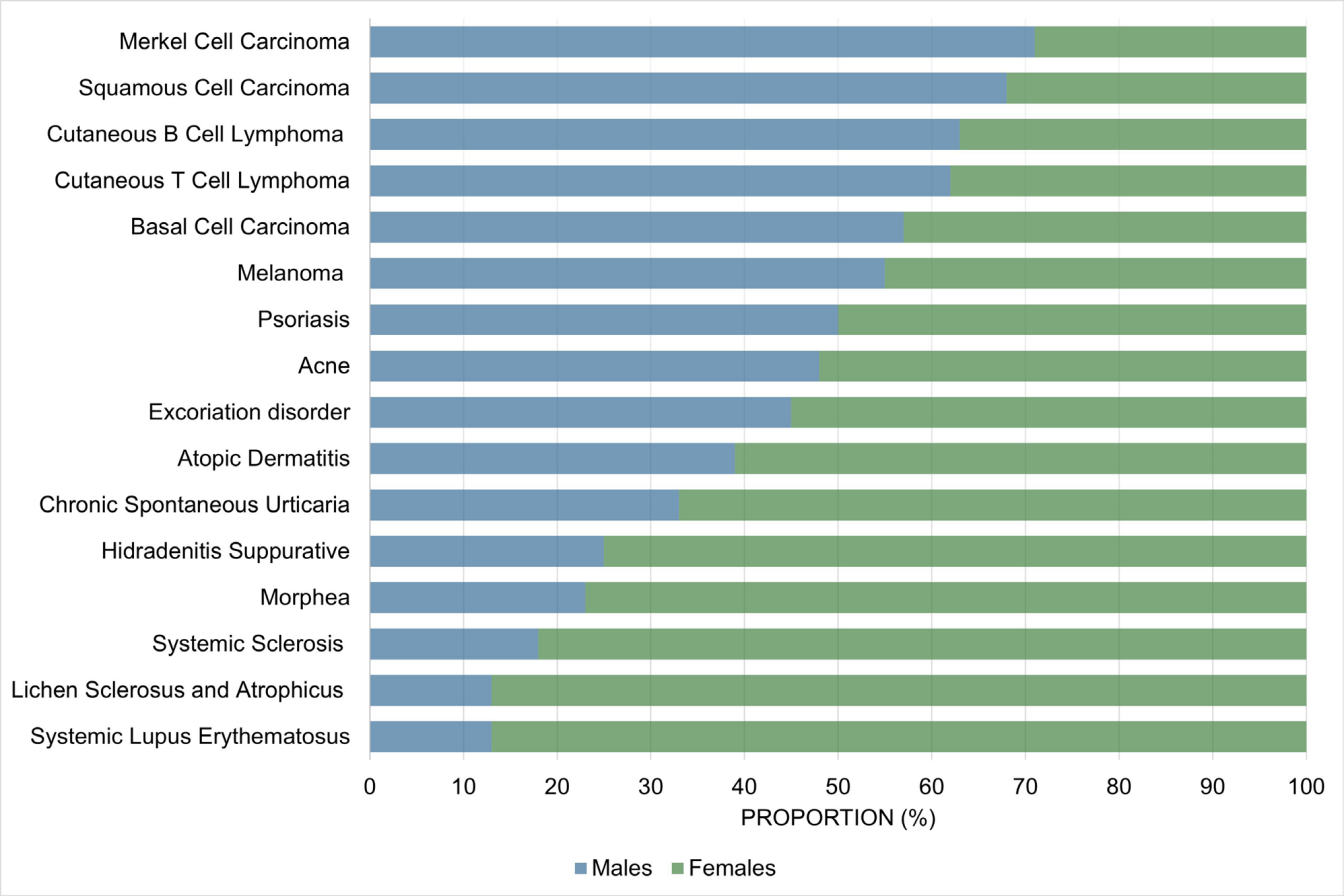
Proportion of male vs. female cases of skin conditions commonly encountered by dermatologists. Trends may differ for specific subpopulations (e.g., children, elderly). For additional details, please refer to Part 2 of the main text.

In this part, we will present the epidemiological and clinical differences observed between males and females in most commonly seen acquired skin diseases in dermatology. In general, psychocutaneous diseases and conditions where autoimmunity plays a major role (e.g., CSU, rheumatologic-dermatologic conditions, and vesiculobullous disorders) show a female predominance. On the other hand, benign and malignant proliferative dermatoses are more common in males. Similarly, paraneoplastic conditions may be more common in males. Other disease categories, such as papulosquamous, lichenoid and eczematous dermatoses, pigmentary disorders, cutaneous adverse drug reactions, infectious diseases, and hair disorders either show no sex/gender predilection or vary depending on the specific disease within the category (e.g., autoimmune alopecia is more common in females *vs*. androgenetic alopecia is more common in males) (Figure 2). We will draw upon concepts presented in Part 1 to discuss possible sex/gender -related factors that could explain the observed differences. Because there is a paucity of studies exploring the impact of gender *vs*. sex in dermatology and the relationship between sex and gender is interconnected and complex, in this section we chose to use the male/female nomenclature regardless of the terms used in the original studies for simplicity.

### Psychocutaneous Diseases

Psychocutaneous disease occurs when skin manifestations are secondary to a primary psychiatric disease and can be divided into delusional, obsessive-compulsive spectrum and related disorders, psychogenic pruritus/pain, factitious, and others.^56^ In general, in adult patients, all psychocutaneous conditions are more common in females, where they represent 55 to 95% of all cases. However, for some conditions, the female-to-male ratio may be equal or reversed in children.^57^ In addition to differences in prevalence, patient’s sex may impact the clinical presentation (*e.g*., extent, distribution, duration), treatment response, as well as the risk/type of comorbid diseases. Table 1 summarizes sex-related differences for the psychocutaneous diseases commonly seen by dermatologists. However, one should be careful when interpreting existing data in psychodermatology as some rates may be overestimated due to bias towards female patients as they are generally more inclined to seek medical attention and more likely to have their medical complaints mislabeled as psychosomatic.^58,59^

**Table 1:** Sex-Gender Related Differences in Psychodermatology.

Disease	Epidemiology differences	Clinical differences	Ref.
**Disorders related to schizophrenia spectrum and other psychotic disorders**			
*Delusion of parasitosis (DP*)	Female predominance with *F*:*M* ratio of ~2:1; sex differences become more pronounced with age (*F*:*M* 3:1 in those over age 50).	Females may have a longer lasting and harder to treat disease.	^139-142^
**Obsessive compulsive and related disorders**			
*Trichotillomania (TTM) and body-focused repetitive behavior disorders (BFRBD, e.g. onychotillomania, onychophagia*)	Up to 77.3 to 94% of all cases occur in females.	Males have an earlier disease onset, account for more childhood cases and commonly present with many comorbidities. In adult male cases, there is a high risk of substance use. Onset is often in adolescence or adulthood for females. Females experience greater impact and disability from TTM.	^143-147^
*Body dysmorphic disorder (BDD*)	Female preponderance in the general population. Similar prevalence in the dermatological and cosmetic setting.	Females express concerns about a larger number of body areas and perform more repetitive behaviors associated to their BDD. Higher rates of concurrent eating disorders were reported. Males are more concerned over their general build, hair thinning, and appearance of genitalia. Males have more concurrent substance use disorders and are more likely to be off work due to their BDD.	^148,149^
*Excoriation disorder (including acné excoriée*)	Approximately 55% of cases are female in the general population, but 75 to 90% of cases are female in the student population and in the dermatology setting. *Acné excoriée* is more common in females (*F*:*M* 10:1).	Legs are the most common affected site in males whereas it’s the face (*e.g., acné excoriée des jeunes filles*) in females. Higher rates of depressive symptoms in females.	^58,145,150-154^
*Psychogenic pruritus and cutaneous sensory disorders (e.g., burning mouth, chronic idiopathic mucocutaneous pain syndromes*)	Female predominance is reported.	N/A	^56^
**Somatic and related disorders**			
*Dermatitis artefacta*	Over 70% of patients are female with a *F*:*M* ratio of 3:1.	Lesion distribution and type differs among sexes with males more likely to report a single ulcer on their leg whereas females are more likely to complain of multiple (often facial) excoriations.	^155-157^
**Other primary psychiatric disorders with cutaneous manifestations**			
*Nonsuicidal self-injury (NSSI*)	More common in females (*F*:*M* 1.5:1).	Females are more likely to choose methods of cutting, scratching, hair pulling and pinching. Males are more likely to choose methods of burning and hitting.	^158^
*Excessive tanning behavior*	Twice more females than males meet criteria for dependence of tanning.	N/A	^159^

Abbreviation: N/A, No data available.

### Papulosquamous, Lichenoid, and Eczematous Diseases

In general, psoriasis prevalence is similar in both sexes. However, in early onset psoriasis, a slight male preponderance has been suggested (male to female ratio of ~1.2 to 1).^60,61^ Disease severity is worse in males, notably male patients are twice as likely to require a systemic treatment^62-65^ and have ~1.5 times higher Psoriasis Area Severity Index (PASI) score for all body sites (except the scalp) than females.^66^ Both sex (e.g., hormonal and immune mediated differences) and gender-related factors (e.g., dietary considerations, sedentarism, smoking, alcohol consumption, prevalence of metabolic diseases) are likely to be important.^67,68^ In addition, trends for special-site psoriasis may be at least partially explained by gender-related practices contributing to koebnerization.^69-71^

Atopic dermatitis (AD) affects female and male children equally but is usually more prevalent in the female population as of puberty.^72-76^ This trend is also observed in other atopic conditions such as asthma and allergic rhinitis.^77^ Gender-related factors including grooming, occupational exposures, cosmetics use, wet work, and stress levels may contribute to this trend.^74^

Differences in allergic (ACD) and irritant contact dermatitis (ICD) prevalence between sexes have not been reported. The clinical presentation may however differ by sex. Head and neck dermatitis is 1.5-2 times more common in females^78-80^ likely associated with gender-related practices such as cosmetics and jewellery use.^80^ Unsurprisingly, the contact allergy to nickel and cosmetics is more prevalent in females.^80,81^ Occupational contact dermatitis is also 50% more common in females and is associated with high risk (wet work) gender-related occupations such as healthcare workers, housekeepers/cleaners, hairdressers, and kitchen workers such as cooks and bakers.^78,82^ Males with contact dermatitis more frequently hold occupations as carpenters, electricians, construction workers, and rubber industry workers.^82,83^ Sex and gender differences regarding the epidemiology of other papulosquamous conditions and lichenoid disorders are summarized in Table 2.

**Table 2:** Epidemiology and Clinical Differences Between Sexes for Papulosquamous, Eczematous, and Lichenoid Dermatoses.

Disease	Epidemiology differences	Clinical differences	Ref.
**Papulosquamous diseases**			
*Psoriasis*	Equal prevalence except possible male predominance in early-onset psoriasis (*M*:*F* 1.2:1). Risk factors and comorbidities of psoriasis are more prevalent in males (cardiovascular disease, smoking, alcohol consumption).	Male sex is a risk factor for severe disease (*M*:*F* 1.4-2.5:1 in severe psoriasis). Worse disease is seen in males for special site psoriasis except scalp psoriasis (equal) and palmoplantar pustulosis (more prevalent in females). Studies in psoriatic arthritis patients show that oligoarthritis and nail involvement is more common in males, while females experience more polyarthritis.	^60-71,160^
*Pityriasis rosea*	Female predominance (*F*:*M* 3:1). More common in pregnant females than in the general population.	N/A	^161-164^
**Eczematous conditions**			
*Atopic dermatitis*	Similar prevalence in children, but female predominance from puberty onwards.	No difference in disease severity. Sleep disturbances due to pruritus more common in females. No significant difference in clinical presentation.	^72,74,77,165,166^
*Contact dermatitis*	Higher prevalence in females. Occupational dermatitis is 1.5-times more common in females and is related to gender risk (2, 5 times) of wet work occupations.	Head and neck dermatitis and allergy to nickel are more prevalent in females and is related to jewelry and cosmeceuticals.	^78-82,83,167,167^
*Seborrheic dermatitis*	More common in males (*M*:*F* 1.2:1). Hypothesized to be related to androgenic activity of sebaceous glands.	N/A	^168-170^
*Venous statis dermatitis*	Higher prevalence of chronic venous insufficiency in females.	N/A	^171^
**Lichenoid diseases**			
*Lichen planus*	More common in females (*F*:*M* 1.2:1). Sex hormones, autoimmunity and stress may be implicated.	Oral lichen planus was reported up to 1.4-2 times more frequently in females.	^172-178^
*Lichen striatus*	Conflicting results regarding sex distribution.	N/A	^179^
*Lichen nitidus*	No sex predilection.	N/A	^180^
*Lichen sclerosus and atrophicus (LSA*)	Overall female predominance (*F*:*M* 1-10:1). Higher prevalence in males during childhood (*M*:*F* 1.7:1). Female disease peaks in prepuberty and post-menopause, whereas male disease peaks in childhood and in the fourth decade of life.	Females are more symptomatic from their disease and are more likely to report concurrent autoimmunity. Extragenital manifestations are more frequent in females. Female children report more cutaneous atrophy and hypopigmentation *vs*. males. Male children more frequently report scarring or changes in genital anatomy. No difference in squamous cell carcinoma prevalence between sexes.	^181-185^

Abbreviation: N/A, No data available.

### Adnexal Diseases

Conditions resulting from dysfunction of hair follicles, sebaceous, eccrine, or apocrine glands are presented under the umbrella of adnexal diseases. Sex/gender considerations of the most common conditions are presented in Table 3. In general, acne vulgaris, rosacea, hidradenitis suppurativa (HS), and hyperhidrosis prevalence is reported to be higher in females; however, the severity and pattern may differ depending on the sex and the specific conditions. Both hormonal (e.g., sex hormones) and gender-related factors (e.g., smoking, obesity, occlusive cosmetics, occupation, likelihood to seek medical attention/treatment, personal/social/cultural perceptions) influence the observed trends.

**Table 3:** Epidemiology and Clinical Differences Between Sexes for Adnexal Diseases.

Disease	Epidemiology differences	Clinical differences	Ref.
*Acne vulgaris*	More common in males during puberty (ages 15-18). More common in females for all other age groups and especially among the adult population.	More severe acne in males (including higher rates of acne fulminans) which can be related to biologic factors (androgens); however, sex/gender-bias should also be considered as females are more likely to consult and receive treatments for acne (all severities) whether prescribed or over the counter. Pattern of skin involvement is generally similar with the exception of chin and lower face predominance in adult-onset female acne. More treatment options are available for female patients such as combined oral contraceptives and anti-androgens, and females respond better to topical dapsone. Greater psychological impact in females.	^22,186-199^
*Rosacea*	Female predominance (~60% overall and ~90% for fulminans) for all subtypes except for phymatous rosacea (more common in males).	Severity depends on the subtype, earlier diagnosis is seen in females, +/-role of gender/sex bias.	^200-207^
*Hidradenitis suppurativa*	*F*:*M* ratio is ~2-4:1 except studies from Asia reporting a male predominance with a *M*:*F* ratio of 2.5:1. There is no difference in the age of onset across sexes.	Different disease pattern and distribution including greater and more severe neck, retroauricular, and buttock disease in males. Greater and more severe axillary and inframammary involvement in females. Males present later in their disease course and have larger wounds that take longer to heal when managed surgically. Higher risk of malignant transformation (Marjolin’s ulcer) in males. Higher rate of sexual distress and more depressive symptoms in females.	^208-220^
*Hyperhidrosis*	While males tend to sweat more physiologically, the epidemiology of hyperhidrosis is conflicting. Clinical data suggests a slight female predominance (~60%) whereas population-based surveys showed no sex difference, suggesting female patients may be more likely to consult medical professionals than males.	No difference in disease severity. Males more frequently present with face and scalp hyperhidrosis in addition to the back, chest, abdomen, forearms, genitals and/or lower extremities. Conflicting evidence regarding the sex predominance of palmar, plantar, and axillary hyperhidrosis. Female patients are more likely to discuss their hyperhidrosis with physicians.	^221-227^

### Urticaria, Eosinophilic and Neutrophilic Dermatoses

Diseases where the role of mast cells, eosinophils and/or neutrophils is predominant are included here. Conditions where autoimmunity plays an important role such as CSU and/or chronic inducible urticaria (CIndU) are female-predominant in adults (similar sex-ratio in children). While neutrophilic diseases (e.g., Behçet’s Disease (BD), Sweet’s Syndrome (SS), Pyoderma Gangrenosum (PG)) have a female predominance except for malignancy-induced cases (equal), mortality may be worse in males. Specific findings are summarized in Table 4.

**Table 4:** Epidemiology and Clinical Differences Between Sexes and Genders for Neutrophilic and Eosinophilic Dermatoses.

Disease	Epidemiology differences	Clinical differences	Ref.
**Urticaria and Angioedema**			
*Chronic spontaneous urticaria (CSU*)	In adults, almost twice as common in females than in males, with an even higher female predilection in autoimmune (Type IIb) CSU. Both sex and gender related factors are likely to contribute including female predilection for autoimmunity (in particular for autoimmune CSU), stress, amongst others. No sex predilection in children nor elderly.	Females are more likely to be diagnosed with concomitant autoimmune diseases and to have clinical/serum markers of autoimmune CSU (e.g., positive autologous serum skin test). Disease flares reported around hormonal life events or treatments (e.g., perimenstrual, pregnancy, oral contraceptives). Female sex is associated with lower disease resolution and higher healthcare cost/number of office and/or emergency visits. Female sex is associated with increased disease severity and a greater prevalence of angioedema.	^228-241^
*Chronic inducible urticaria (CIndU*)	Male predominance seen with cholinergic urticaria. Female predominance seen with cold urticaria in adults (not children) and symptomatic dermographism with a *F*:*M* ratio 1.3-1.4:1. Sex-disaggregated data of other types of CIndU (delayed pressure, solar, aquagenic and heat urticaria) are insufficient to draw conclusions.	Female sex is associated with lower disease resolution. Females with cholinergic urticaria have a later age of onset.	^231,236,242-246^
*Hereditary angioedema (HAE) and acquired angioedema (AAE*)	HAE types I and II do not have any sex predominance. HAE type III and angiotensin converting enzyme inhibitor- induced AAE show a female predominance.	Females with HAE have more episodes per year than males. Oral contraceptives, estrogen replacement therapy, and pregnancy may trigger or worsen symptoms in HAE. In HAE types I and II, females report increased HAE-related stress and fear compared to males.	^247-253^
**Neutrophilic Dermatoses**			
*Behçet’s disease (BD*)	No sex predilection.	Earlier age of onset in females (thirties *vs*. fifties in males). Female sex increases the risk of erythema nodosum, genital ulcers, and joint involvement. Male sex increases the risk of ocular involvement, folliculitis/papulopustular skin lesions, deep venous and superficial thrombosis, and pathergy test positivity. Up to 5-fold mortality risk increase in males.	^254-256^
*Pyoderma gangrenosum (PG*)	Limited data suggests higher prevalence in females (up to 2-fold); however, a similar incidence between sexes was reported.	N/A	^257,258^
*Sweet’s syndrome (SS*)	More common in females (*F*:*M* 3-6:1) for the idiopathic and drug-induced subtypes. No sex predisposition in cancer-related cases. Because of pregnancy induced SS cases, hormonal influences may be of importance.	N/A	^259-266^
*Synovitis, acne, pustulosis, hyperostosis and osteitis syndrome (SAPHO*)	Female predominance, especially in children.	Female sex is associated with a more chronic disease course.	^267-269^
**Eosinophilic Dermatoses**			
*Granuloma faciale*	Male predominance (*M*:*F* 1.7-2.1:1).	Mean age of onset is comparable between males and females.	^270-272^
*Papuloerythroderma of Ofuji*	Male predominance in infancy and in the immunosuppressed, but not the classic variant.	Younger age of onset in females. More facial involvement in females than males. More males complain of associated pruritus.	^273-276^
*Wells Syndrome*	No known sex predilection.	N/A	^277^
*Hypereosinophilic syndrome*	Male predominance.	Patients with *FIP1L1-PDGFRA* fusion gene positive chronic eosinophilic leukemia are almost exclusively male.	^278-280^
*Erythema annulare centrifugum*	Unclear sex predilection.	Similar age of onset between males and females.	^281-283^

Abbreviation: N/A, No data available.

### Connective Tissue Diseases

Most autoimmune diseases have a higher predilection for females than males.^7^ While up to 90% of patients diagnosed with systemic lupus erythematosus (SLE) are female, a similar predominance is seen in cutaneous lupus erythematosus (CLE), systemic sclerosis (SSc), morphea, dermatomyositis, and Sjogren’s syndrome.^84-96^ Interestingly, in general, males have a higher disease severity, greater prevalence of organ involvement and increased mortality.^97-101^ It is hypothesized that a combination of genetic, hormonal, and environmental influences (as discussed in Part 1) accounts for the female predominance and the differences in clinical presentation between the sexes.^7,102-104^ Other environmental factors such as infectious pathogens, diet, exposure to chemicals/toxins and UV light, stress and hormonal therapy vary between males and females and are linked to the development of autoimmune disease.^7,103,105,106^ Specific findings for connective tissue diseases are presented in Table 5.

**Table 5:** Epidemiology and Clinical Differences Between Sexes and Genders for Connective Tissue Diseases.

Disease	Epidemiology differences	Clinical differences	Ref.
**Systemic Autoimmune Rheumatic Diseases (SARD)s**			
*Systemic lupus erythematosus (SLE*)	There is a female predominance in every age group. The *F*:*M* ratio is 2-6:1 before puberty, 8-15:1 in reproductive years, and 3-8:1 after menopause. Males account for approximately 4 to 22% of SLE cases. There is evidence that males with SLE have a higher number of risk alleles than females, suggesting that males need a greater cumulative genetic burden to develop the disease.	Males are older at diagnosis, have more frequent renal disease, skin involvement, hematological disease, serositis, neurological disease, vascular thrombosis, cardiovascular complications, hepatosplenomegaly, fever and weight loss at disease onset, neoplasms, hypertension, and vasculitis. Male sex is a risk factor for mortality. In males, discoid lesions and/or subacute lesions are more common, and malar rash is less common. Males have less frequent Raynaud phenomenon, photosensitivity, mucosal ulcers, lymphadenopathy, and thyroid disease than females. Possible decreased anti-Ro (or SSA) and anti-La (or SSB) antibodies, low complement component 3, low CH50, and higher frequency of anti-dsDNA, anti-Sm antibodies, anticardiolipin antibodies, anti-U1 nuclear ribonucleo- protein (U1RNP) antibodies and lupus anticoagulant in males.	^97,284^
*Systemic sclerosis (SSc*)	Higher prevalence in females (*F*:*M* ~ 3–8:1).	Diagnostic delay (time from first non-Raynaud phenomenon until clinical diagnosis) is longer in females. Females have a higher frequency of limited cutaneous SSc and associated features, notably peripheral vascular disease, anticentromere antibody (ACA), anti-topoisomerase I and anti-U3RNP antibody positivity. Males have higher frequency of diffuse SSc, interstitial lung disease and mortality. Most common cause of death in males and females is interstitial lung disease and pulmonary hypertension, respectively. Occupational exposure to silica, organic solvents, and cigarette smoking are more frequently reported in males with SSc and contribute to a more severe disease phenotype and poor survival.	^86,98,99,106^
*Sjogren’s syndrome*	Female predominance (*F*:*M* 9-14:1).	Males have a higher frequency of extra-glandular manifestations such as pulmonary involvement, vasculitis, and lymphadenopathy. Males are younger at diagnosis and have more interstitial lung disease and cutaneous vasculitis than females. Males who are SSA-positive have higher levels of anti-Ro52 than females.	^100^
*Dermatomyositis*	Higher incidence in females (*F*:*M* ~ 2–3:1).	Possible increased risk of malignancy in males.	^101,285,286^
**Other Autoimmune/Rheumatic Diseases**			
*Still’s disease*	2-fold higher prevalence in females than males in the United States. In the elderly population, more common in females (*F*:*M* is 4:1). Equal distribution between sexes in Europe.	N/A	^287-291^
*Relapsing polychondritis (RPC*)	Most studies suggest no sex predominance; however, some studies report *F*:*M* ratio 1.5-3:1.	Males more frequently have myelodysplastic syndrome. Anecdotally, higher frequency of hearing loss, uveitis, vestibular disease, hypertension, diabetes mellitus was observed in males. Worse prognosis is generally reported in males. Females have higher rates of concomitant autoimmunity (e.g., SLE, other SARDs, thyroid autoimmune disease, etc.). No sex difference in dermatological symptoms.	^292-295^
*Familial Mediterranean fever (FMF*)	N/A	Earlier age at diagnosis in males. Anxiety, depression, migraines, and headaches are more common in females. No sex difference in disease severity.	^296^
*Morphea (localized scleroderma*)	Female predominance (*F*:*M* 2.4-4.2:1).	Female sex is a significant predictor of impaired health-related quality of life.	^297-299^
**Vasculitides**			
*Cutaneous small vessel vasculitis*	Similar incidence between sexes.	Male sex is not a significant predictive factor for disease relapse.	^300,301^
*Small/ small and medium vessel vasculitis*	Henoch-Schoenlein purpura (HSP) shows a male predominance below age 10, but a female predominance afterwards. Cryoglobulinemic and hypocomplementemic urticarial vasculitis have a female predominance. ANCA-associated vasculitis has no sex predominance in adults, but a female predilection in children.	No reported differences for HSP, cryoglobulinemic vasculitis, and hypocomplementemic urticarial vasculitis. In ANCA-associated vasculitis, male sex is a risk factor for all-cause mortality, and may be a risk factor for progression to end-stage renal disease.	^300,302-308^
*Medium vessel vasculitis*	Male predominance for Kawasaki disease. Conflicting data on sex predilection for polyarteritis nodosa.	In Kawasaki disease, severe cardiac complications are more common in males. No reported differences for polyarteritis nodosa.	^309-313^
*Large vessel vasculitis*	Female predominance for Takayasu’s and giant cell arteritis.	Females with Takayasu’s arteritis have a lower age of onset and a lower incidence of cardiac complications compared to males. In giant cell arteritis, males have an increased risk of aortic aneurysm and eye involvement, whereas females have a higher risk of jaw involvement and polymyalgia.	^314-320^

Abbreviation: N/A, No data available.

### Pigmentary Disorders

Pigmentary disorders comprise de-, hypo- and hyperpigmentation. In this section, we will present the sex and gender differences seen in vitiligo, pityriasis alba, progressive macular hypomelanosis, idiopathic guttate hypomelanosis, post-inflammatory hypo- and hyperpigmentation, melasma, erythema dyschromicum perstans, and prurigo pigmentosa (Table 6). In general, beside vitiligo, data is scarce and hence, strong conclusions can not be drawn.

**Table 6:** Epidemiology and Clinical Differences Between Sexes and Genders for Pigmentary Disorders.

Disease	Epidemiology differences	Clinical differences	Ref.
**Depigmented and hypopigmented conditions**			
*Vitiligo*	Multiple studies have shown no significant sex predominance.	Females have more frequent involvement of the trunk, hips, groin, arms, elbows, feet, and axilla. Males have more frequent involvement of the hands, fingertips, and genitals (lip-tip vitiligo), as well as leucotrichia. On the face, females have more periocular involvement, while males have more involvement at the beard area. Vitiligo is known to be induced by Koebner’s phenomenon; therefore, the documented sex differences may in fact be behavior-related (e.g., hair removal, cosmetic practices).	^321-323^
*Pityriasis alba*	Male predominance.	N/A	^324^
*Progressive macular hypomelanosis*	Possibly higher prevalence in females based on 1 study (*F*:*M* ~ 7:1).	N/A	^325^
*Post-inflammatory hypopigmentation*	No difference in incidence by sex.		^326^
*Idiopathic guttate hypomelanosis*	No sex predilection.	Possible earlier onset in females.	^327^
**Hyperpigmented conditions**			
*Melasma*	Higher prevalence in females (*F*:*M* 1.2-10:1). Increased incidence in pregnant females and those on oral contraceptives or hormonal replacement therapy.	Females more frequently display centro-facial pattern of involvement. Males more frequently display malar involvement.	^328-333^
*Erythema dyschromicum perstans*	Possible female predominance based on 1 study.	N/A	^334^
*Prurigo pigmentosa*	Higher prevalence in females (*F*:*M* 2:1). Prurigo pigmentosa has been linked to restrictive ketogenic diets; however, it remains unclear if this diet is more commonly adopted by females.	N/A	^335,336^
*Post-inflammatory hyperpigmentation*	No sex predilection.	N/A	^337^

Abbreviation: N/A, No data available.

### Immune-Mediated Vesiculobullous Diseases

In general, vesiculobullous diseases either have a female predominance, likely due to the autoimmune nature of many of these diseases, or no clear sex predilection. Possibly, paraneoplastic immunobullous diseases are more common in males. Diseases in this category are summarized in Table 7.

**Table 7:** Epidemiology and Clinical Differences Between Sexes and Genders for Cutaneous Vesiculobullous Diseases.

Skin condition	Epidemiology differences	Clinical differences	Ref.
*Pemphigoid*	Conflicting evidence, but most studies suggest a female predominance in bullous pemphigoid (*F*:*M* 1.0-5.1:1). Female predominance in mucous membrane pemphigoid (*F*:*M* 4.3:1).	No difference between sexes in mortality rates or overall survival.	^338-342^
*Pemphigus*	Overall female predominance (*F*:*M* 1-5:1) for Pemphigus Vulgaris (PV). Conflicting results or equal sex distribution in Pemphigus Foliaceus (PF) and Herpetiform Pemphigus. Possibly higher risk of paraneoplastic pemphigus in males.	Males with PV are more likely to develop the disease before age 40, have more widespread cutaneous involvement, and express antibodies to both desmoglein-1 and desmoglein-3. Females with PV have more mucosal involvement and are more likely to have a personal or family history of autoimmune diseases. Sex does not have an impact on overall survival in PV or PF.	^343-348^
*Dermatitis herpetiformis*	No clear sex predilection.	Female sex is associated with delayed diagnosis, and males are diagnosed at an older age than females. Together, these findings could suggest an earlier onset of symptoms in females. Concomitant autoimmune thyroid disease is more common in females.	^349-351^
*Linear IgA bullous dermatosis*	N/A	N/A	^352^

Abbreviations: BP, bullous pemphigoid; N/A, No data available; PF, pemphigus foliaceus; PV, pemphigus vulgaris; RR, relative risk.

### Cutaneous Adverse Drug Reactions

Overall, there is very limited data on sex and gender differences in adverse cutaneous drug reactions. Many epidemiology studies looking at Steven-Johnson Syndrome (SJS)/Toxic Epidermal Necrolysis (TEN) and drug reaction with eosinophilia and systemic symptoms (DRESS) have shown that females are overrepresented.^107-113^ For acute generalized exanthematous pustulosis (AGEP), generalized bullous fixed drug eruption (GBFDE), and exanthematous drug eruptions, there is no significant sex predominance or differences in clinical presentation that has been revealed conclusively by previous epidemiologic studies or case series.^114-120^ Table 8 summarizes the findings for cutaneous adverse drug reactions.

**Table 8:** Epidemiology and Clinical Differences Between Sexes and Genders for Cutaneous Adverse Drug Reactions.

Skin condition	Epidemiology differences	Clinical differences	Ref.
*Steven-Johnson Syndrome (SJS)/Toxic Epidermal Necrolysis (TEN*)	Overall female predominance.	More females hospitalized for TEN (*F*:*M* 2-2.6:1). More males hospitalized for SJS (*M*:*F* 1.6-2:1).	^107-353,354,354^
*Drug reaction with eosinophilia and systemic symptoms (DRESS*)	Some studies report higher prevalence in females.	N/A	,^110-113,355^
*Acute generalized exanthematous pustulosis (AGEP*)	No significant sex predominance has been reported.	N/A	^114-120^
*Generalized bullous fixed drug eruption (GBFDE*)	No significant sex predominance has been reported.	N/A	
*Exanthematous drug eruption*	No significant sex predominance has been reported.	N/A	

Abbreviation: N/A, No data available.

### Infectious Diseases

For the scope of this review article, we focus on infectious diseases most commonly seen in clinical practice. In general, studies have shown that males are more susceptible to infections than females. As detailed in part 1, this may be attributable to an overall heightened immunity in females.^2^ Gender-specific recreational and occupational activities can also lead to exposure to different types of pathogens, which could contribute to the observed differences.^2,8,121^ Sex- and gender-related differences for infectious diseases are reviewed in Table 9.

**Table 9:** Epidemiology and Clinical Differences Between Sexes and Genders for Cutaneous Infectious Diseases.

Skin condition	Epidemiology differences	Clinical differences	Ref.
**Bacterial infections**			
*Bacterial cellulitis*	Higher incidence in males (*M*:*F* 1.0-2.4:1) for both community- and hospital-acquired disease.	Females with cellulitis are older and usually have venous insufficiency or lymphedema as underlying conditions. Males are more likely to develop cellulitis following a wound. The most frequent location in both sexes is the lower extremities.	^356-359^
*Lyme disease*	Conflicting evidence, but possible higher incidence in males, may be related to gender-specific hobbies and exposures.	No difference between sexes in the staging at presentation. Females with Lyme disease are older. Females are more likely to be diagnosed during low-season months.	^360,361^
*Syphilis*	Higher incidence and prevalence in males, especially among males who have sex with males (MSM).	Higher mortality rates in males.	^362,363^
***Viral infections***			
*Herpes zoster*	Overall higher risk in females (OR1.3, RR 1.4).	More females develop post-herpetic neuralgia.	^364-367^
*Herpes simplex virus (HSV) 1 and 2*	HSV-1 and HSV-2 are overall more common in females.	Systemic symptoms and complications (*e.g*., aseptic meningitis) more common in females. Commonly affected sites for females include the oropharynx, labia majora, labia minora, mons pubis, vaginal mucosa, buttock, and cervix. Commonly affected sites for males include the oropharynx and shaft of the penis. HSV-2 is more transmissible sexually from males to females than females to males. Herpes gladiatorum is seen in young athletes that play contact sports, especially wrestling.	^368-372^
*Condyloma acuminatum*	Conflicting evidence, but most studies report slight male predominance (*M*:*F* 0.9-1.3:1). Higher prevalence among MSM.	Males have slightly more frequent and longer duration of episodes than females. Peak occurrence of anogenital warts is later in life for males than females. Cost and resource utilization per disease episode are higher in males. HPV vaccine uptake is significantly higher in females than in males.	^373-381^
*Warts (Verruca plana, vulgaris, and plantaris*)	No sex predilection in children. Verruca vulgaris and plantaris show a male predilection in adults. Limited data is available on verruca plana in adults	N/A	^382-385^
*Human immunodeficiency virus (HIV*)	In North America, higher prevalence and incidence in males, especially MSM	Males have an increased risk for late disease presentation, progression to acquired immunodeficiency syndrome, and death.	^386-389^
*Human herpesvirus 8 (HHV8*)	No sex predominance of HHV8 in children or in adults. HHV8 seroprevalence is higher among MSM.	Data on primary HHV8 infection in HIV negative populations are scarce. HIV co-infected males are at increased risk of associated Kaposi’s sarcoma.	^390-392^
*Molluscum contagiosum*	No sex predominance.	N/A	^393,394^
**Fungal infections**			
*Dermatophytes*	Male predominance.	N/A	^395^
*Candidiasis*	Female predominance.	Common locations for females include intertriginous areas and the vulva.	^395^
**Bites and infestations**			
*Scabies*	Increased risk in females (RR 1.24).	N/A	^396^

Abbreviation: N/A, No data available.

### Cutaneous Neoplasms

Overall, males display higher incidence rates for most types of neoplasms, including many cutaneous neoplasms.^122^ Multiple biological and sociocultural factors likely play a role in the observed sexual dimorphism, as discussed in Part 1. In particular, both genders exhibit different health behaviors: females are more likely to engage in sun protective practices and tend to seek medical attention sooner, resulting in earlier skin cancer detection.^2^ Occupational factors and clothing choices also differ, whereby males are more likely to work outdoors (increased sun exposure on the head/neck and trunk) and females tend to expose their skin to the sun because they perceive a tanned appearance as desirable.^123^ Whereas UV radiation is a well-known risk factor for melanoma, squamous cell carcinoma (SCC), and basal cell carcinoma (BCC), risk factors for developing cutaneous T-cell lymphoma include hydrochlorothiazide diuretic use, immunosuppression, bacterial and viral infections, air pollution, chemical exposures and detergents, amongst others, which can also vary by sex/gender.^124^ Additional information on various cutaneous neoplasms is presented in Table 10.

**Table 10:** Epidemiology and Clinical Differences Between Sexes and Genders for Cutaneous Neoplasms.

Skin condition	Epidemiology differences	Clinical differences	Ref.
*Cutaneous melanoma*	Males have an overall higher incidence (*M*:*F* 1.1-1.3:1). Females have a higher incidence before age 39 and after age 80. Most cutaneous melanoma subtypes except for mucosal lentiginous melanoma and acral lentiginous melanoma are more common in males.	Most common location is on the head, neck, and trunk in males. Males are diagnosed at a more advanced age than females. Most common location is on the extremities in females. Earlier diagnosis, thinner Breslow thickness, less metastases and increased survival in females. No significant association between sex and BRAF mutations.	^397-404^
*Squamous cell carcinoma*	Overall higher incidence in males (*M*:*F* 1.2-2.4:1). Higher incidence in females before age 50 (*F*:*M* 1.3:1).	Most common location is the head and neck in both sexes. No significant difference in the degree of differentiation or tumor size. Local recurrence, nodal metastasis, distant metastasis, and death more common in males.	^405-411^
*Basal cell carcinoma*	Overall higher incidence in males (*M*:*F* 1.1-4.4:1). Higher incidence in females before age 50 (*F*:*M* 1.4:1).	Most common location is the head and neck in both sexes. Most common subtype is nodular in both sexes. No difference in frequency of aggressive subtypes (infiltrating, micronodular, metatypical, morpheaform) between sexes. Larger tumors in males.	^405-412,410-412^
*Merkel cell carcinoma*	Overall higher incidence in males (*M*:*F* 1.4-2.5:1).	Most common location is the head followed by the arms in both sexes. Higher incidence of regional and distant metastases in males. Higher relative survival rates in females.	^410,413-415^
*Cutaneous T-cell lymphoma*	Overall higher incidence in males (*M*:*F* 1.4-1.9:1).	Males have a higher age at diagnosis, especially for mycosis fungoides. Females with mycosis fungoides present more often with lesions on the trunk. Males have higher odds of presenting with a higher T-stage (T3-T4). Females have a better overall survival and disease-specific survival. No difference between males *vs*. females regarding the risk of large plaque parapsoriasis progressing to mycosis fungoides.	^124,416-421^
*Cutaneous B-cell lymphoma*	Overall higher incidence in males (*M*:*F* 1.72:1).	Females have a higher age at diagnosis.	^417^
*Kaposi sarcoma (KS*)	Male predominance (classic KS *M*:*F* 17:1; endemic KS *M*:*F* 2:1; iatrogenic KS *M*:*F* 3:1) AIDS-related KS is almost exclusively seen in males in North America, especially MSM; however, its prevalence has decreased with the introduction of highly active antiretroviral therapy (HAART).	One study from subSaharan African found that HIV-infected females are more likely to present with lesions on the face and hard palate. Males are more likely to have lower extremity lesions. Another study from Tanzania found that males and females are more likely to have localized and disseminated lesions, respectively.	^422-425^
*Dermatofibrosarcoma protuberans (DFSP*)	Conflicting evidence, but most studies report a similar distribution between sexes.	No significant difference between sexes in mean age at diagnosis. Most common anatomic site is the trunk in both sexes. Higher mortality in males. Pregnancy can lead to tumor enlargement or change in color.	^426-430^

### Hair Diseases

This section will review the epidemiological and clinical differences between sexes and genders for various hair conditions. Androgenic alopecia, which is also known as male- and female-pattern hair loss, is more common in males overall. Its the prevalence increases with age in both sexes with 17% and 74% of males ages 20-29 and 80+, respectively, being affected compared to 12% and 57% of females.^125^ In general, apart from AA, hair loss caused by an autoimmune disorder (*e.g.,* frontal fibrosing alopecia, central centrifuging scarring alopecia, lichen planopilaris) affects females more than males. Telogen effluvium is also more common in females. These findings are possibly biased since females are more likely to consult a dermatologist for hair loss.^126^ Further, these conditions have a greater impact on the quality of life of females compared to males.^126,127^ Additional information is presented in Table 11.

**Table 11:** Epidemiology and Clinical Differences Between Sexes and Genders for Hair Diseases.

Hair condition	Epidemiology differences	Clinical differences	Ref.
*Androgenetic alopecia*	Occurs more frequently in males, although it is the most common cause of hair loss in females. Increased incidence in females after menopause.	Males most commonly develop frontoparietal and frontal recession with subsequent vertex thinning. Females most commonly develop diffuse central thinning of the crown with preservation of the frontal hairline.	^431-433^
*Telogen Effluvium (TE*)	More common in females. TE is possibly triggered by stress (*e.g.,* systemic, psychological) and endocrine disorders/changes (*e.g.,* hypothyroidism, hyperthyroidism, post-partum state). Many of these triggers are more common in females.	N/A	^434-436^
*Alopecia areata (AA*)	Conflicting evidence, especially from international studies, which is likely attributable to cultural and religious factors.	In males, the most common complaint is AA of the beard. Males are more likely to be diagnosed in childhood and to have a positive family history. Females have a longer disease duration, are more likely to have nail involvement and a concomitant autoimmune and/or mental health disorder. Conflicting evidence regarding disease severity by sex.	^437-441^
*Central centrifugal cicatricial alopecia*	More common in females, especially African American females. Gender differences, including cultural hairstyling practices, contribute to the female predominance.	N/A	^442,443^
*Lichen planopilaris (LPP) and frontal fibrosing alopecia (FFA*)	More common in females (LPP *F*:*M* 4.9:1; FFA *F*:*M* 31:1).	Disease onset later in females. Similar clinical and histologic features in both sexes.	^444,445^
*Folliculitis decalvans*	Male predominance across multiple studies.	Later age of onset in females than males (42 years vs. 31 years). Males present more frequently with pustules and are more likely to have associated androgenic alopecia.	^446^
*Dissecting cellulitis of the scalp*	Male predominance.	N/A	^447,448^

Abbreviation: N/A, No data available.

## Part 3 – Dermatology in the Transgender Population

In recent years, there has been an increased awareness of the wide range of gender identities within our population. In particular, many individuals’ gender identity is incongruent with their assigned sex at birth. This population includes the transgender community, which encompasses individuals that have a fixed gender identity that is different from their assigned sex at birth, gender fluid individuals whose gender identity is not fixed, as well as nonbinary individuals who do not identify as a man or a woman.^128^ Two spirit is a term used in some Indigenous communities that describes individuals who embody both a masculine and a feminine spirit. In the United States, it is estimated that 0.2 to 2.7% of the adult population identifies as transgender.^128-130^ These individuals may undergo a social, legal, and/or medical transition.^131^

The medical transitioning process can involve hormonal therapy and/or various procedures and surgeries to have a physical appearance that is congruent with one’s gender identity.^128,131^ As such, one’s transition often requires a multidisciplinary approach. Many gender-affirming procedures can be performed by dermatologists, including botulinum toxin injections, cosmetic filler injections, scar revisions from prior surgeries and laser hair removal.^128^ In addition, hormonal therapy inevitably affects the integumentary system and can have an impact on the prevalence and severity of many dermatologic conditions.^132,133^ A list of skin conditions that may be overrepresented in transgender women and men is presented in Supplemental Table 6.

Transgender women are often prescribed exogenous estrogen and antiandrogen therapy to gain a more traditionally feminine appearance. These therapies lead to decreased facial and body hair growth density, decreased sebum production, as well as promote epidermal thickness, fat redistribution, collagen production, and increased melanocyte stimulation.^128,131,132^ In turn, these treatments also have dermatological implications such as increased xerosis and hair/nail fragility, risk of asteatotic dermatitis and melasma, as well as a positive correlation with HIV-related dermatoses.^128,132,133^ In addition, lichen sclerosus and HPV infections (and related skin diseases) have been reported in neovaginas.^132,133^ On the other hand, exogenous estrogen and antiandrogen therapy may improve acne.^128^

Similarly, transgender men often undergo medical treatment to have a more traditionally masculine appearance. For example, exogenous testosterone therapy increases body and facial hair growth, decreases scalp hair, redistributes adipose tissue, and increases sebum production, which can help achieve this goal.^128,132^ Such therapies can have numerous dermatological implications, such as an increased risk of androgenic alopecia and acne vulgaris. In fact, among transgender men receiving masculinizing hormone therapy (MHT), the prevalence of acne increases from 6.3% to 31.1%.^134^ Androgen therapy has also been associated with an increased risk of HIV-related dermatoses.^128,133^

Treating the transgender population requires the clinician to be mindful of numerous bioethical principles. For example, acne (often severe) in transgender men may necessitate systemic therapy with isotretinoin. There are numerous bioethical factors to consider when prescribing isotretinoin, a highly teratogenic agent, to a transgender man that may still have native internal female reproductive organs. Treating physicians should also be aware that some transgender men with native internal female reproductive organs desire a planned pregnancy.^135^ *Richer et al* have recommended respectfully addressing reproductive status, anatomy, and sexual practices to gauge pregnancy risk, guide counselling, and provide the best care for these patients.^136^ For example, it is important to consider that a transgender man may no longer have a uterus or may engage in sexual activities with individuals who do not have a penis and/or testes with reproductive potential.^136^ Previously, the iPLEDGE program (United States of America) required clinicians to categorize patients as either: male, female of nonchildbearing potential, or female of childbearing potential and only the latter had to undergo strict pregnancy monitoring. This classification system posed a significant dilemma for patients and clinicians who had to either categorize the patient according to their assigned sex at birth (and neglect the patient’s identity) or based on their gender identity (which did not respect prior iPLEDGE principles). Many groups have advocated to change the classification system in order to respect patient autonomy,^137^ and as a result, starting at the end of 2021, the iPLEDGE program reduced the patient risk categories from three options to two: patients who can get pregnant and patients who cannot get pregnant. This change has the potential to help bridge the gap in transgender health services. A comprehensive review on dermatological care in transgender individuals is discussed elsewhere.^128,138^

## Conclusions and Future Directions

In conclusion, it is imperative for dermatologists and practicing clinicians to distinguish sex from gender and to recognize both as distinct broad risk factor categories for skin diseases. In this review, we highlight the sex-related differences between males and females, as well as the gender-related differences between men and women. We examine epidemiological and clinical differences between sexes and genders for numerous dermatological disease categories and proposed sex- and gender-related factors that could explain the observed differences. We also discuss populations where there is incongruence between the assigned sex at birth and the gender identity of an individual. Together, these discussions highlight the importance of studying sex, gender, and gender identity separately, as well as identifying and studying their intersections, instead of viewing these epidemiological categories as 1 single entity.

This article is a narrative review and has intrinsic limitations. First, reviews are subject to publication bias, where articles that report significant results are more likely to be published and therefore more likely to be included in reviews than studies that do not. In addition, a systematic review was not performed given the broadness of the chosen topic. Therefore, there may be an element of selection bias regarding the articles that were included in the review. Second, this study did not include all disease categories. For example, genital diseases and genodermatoses were not included, and are outside of the scope of this article. Third, there has been little research conducted with transgender populations to indicate to what extent they follow the gender norms and health-related habits presumed to be associated to their gender identity. Lastly, the impact of gender-affirming treatments and procedures on the integumentary system is an area of research which requires further exploration.

Through this article, we hope to highlight that there are many areas within the field of dermatology that can still be improved to ensure a more comprehensive patient-centered care and to provide the best care for all patients. It is important to practice compassionate and inclusive medicine, and to recognize that the population is becoming increasingly diverse. Strategies to provide a more inclusive practice could include asking one’s preferred name and pronouns, as well as using inclusive language that does not assume one’s gender identity or sexual orientation.

## Supplemental Material

Supplementary material - Supplemental material for The Role of Sex and Gender in Dermatology - From Pathogenesis to Clinical Implications Click here for additional data file. Supplemental material, Supplementary material, for The Role of Sex and Gender in Dermatology - From Pathogenesis to Clinical Implications by François Lagacé, Kathleen D’Aguanno, Connor Prosty, Alexandra Laverde-Saad, Leila Cattelan, Lydia Ouchene, Sarah Oliel, Genevieve Genest, Philip Doiron, Vincent Richer, Abdulhadi Jfri, Elizabeth O’Brien, Philippe Lefrançois, Mathieu Powell, Linda Moreau, Ivan V. Litvinov, Anastasiya Muntyanu and Elena Netchiporouk in Journal of Cutaneous Medicine and Surgery
