# Supplementary material for: The Role of Sex and Gender in Dermatology - From Pathogenesis to Clinical Implications
Source: J Cutan Med Surg. 2023 Jul 4;27(4):NP1–NP36. doi: 10.1177/12034754231177582 (PMC10486181; doi:10.1177/12034754231177582)
Supplement: Supplementary material - Supplemental material for The Role of Sex and Gender in Dermatology - From Pathogenesis to Clinical Implications [file sj-pdf-1-cms-10.1177_12034754231177582.pdf]

Role of sex- and gender- related risk factors in dermatological diseases

Supplementary file

Sex hormones and embryogenesis

At fertilization, the sex of the embryo is determined based on the sexual chromosomes. Multiple steps then take place during which sexual organs acquire male or female characteristics (449). To illustrate, the presence of the SRY gene (and other genes on the Y chromosome) promotes the testicular pathway and inhibits the ovarian pathway (12, 449). Once the developing gonad undertakes either the testis or the ovary pathway, it produces molecules and hormones that facilitate the subsequent development of the associated reproductive organs (450). The presence or absence androgens and anti-Müllerian hormone determines if the internal and external genitalia follow the male or female pathway, respectively (449). If these conditions are present, the Müllerian duct will regress, and the Wolffian ducts will form the epididymis, vas deferens, and seminal vesicles, and the urogenital sinus will give rise to the prostate and bulbourethral glands, thus forming the internal male reproductive organs (450). The external male genital organs, such as the glans penis, penile shaft, urethra, prepuce, and scrotum will arise from the genital tubercle, glans, urethral folds and labio-scrotal bulges, a process that is driven by the conversion of testosterone to dihydrotestosterone (450). On the other hand, if these conditions are absent, the Müllerian ducts will become the fallopian tubes, uterus, cervix, and upper part of the vagina, and the urogenital sinus will form the lower region of the vagina, giving rise to the internal female reproductive organs (450). Female external genital development, including the formation of the clitoris, labia minora, and labia majora from the genital tubercle, urethral folds, and labioscrotal folds, is mediated by estrogens (450).The importance of hormonal function in gonadal

development and body habitus is illustrated by complete androgen insensitivity syndrome (CAIS). Individuals with CAIS are genotypically 46XY but due to androgen receptor mutation, have profound resistance to androgens. Due to androgen resistance, but preserved androgen to estrogen conversion through the action of aromatase, these patients phenotypically develop as females. Patients with CAIS develop female external genitalia, breast, and female-pattern body habitus that are non-ambiguous in appearance with little to absent axillary/pubis hair and higher than expected for female stature. They are usually raised and self-identify as women and the diagnosis is typically made during adolescence or young adulthood due to primary amenorrhea or infertility. Given their 46XY genotype, these patients do not have ovaries or uterus, but rather non-ambiguous undescended testes (451, 452).

Supplementary Table 1. Effects of estrogen on the immune system

**Cell type**	**Function**	**Effect**	**Ref**
Dendritic cells	Antigen presentation and pro-inflammatory cytokine release	↑	(8, 26, 453, 454)
	Differentiation and maturation of epidermal Langerhans cells	↑	
Monocytes	Adhesion to endothelium	↓	(6)
Tregs	Differentiation and function	↑**	(6)
CD4+ T cells	Th1 immune pathway from CD4+ T cells (including TNF-alpha, IL-1-beta, TGF-beta)	↑*<br>↓**	(6-8)
	Th2 anti-inflammatory pathways (including IL-4, IL-10, TGF-beta)	↑**	
CD8+ T cells	Cytotoxic effects of TNF-alpha	↓	(7)
	CD4+/CD8+ T cell ratio	↓*	
B cells	B cell activation and survival	↑*	(6, 7, 26)
	Immunoglobulin secretion	↑*	

*At low concentrations of estrogen
**At high concentrations of estrogen

Supplementary Table 2. Effects of progesterone on the immune system

Cell type	Function	Effect	Ref
Thymocytes	Apoptosis	↓	(455)
Granulocytes	Apoptosis, superoxide release and chemotaxis from neutrophils	↓	(247)
	Degranulation from eosinophils	↑	
NK cells	Apoptosis	↑	(7, 247)
	Cytotoxicity and IFN-gamma release	↓	
Macrophages	Expression of toll-like receptors	↓	(6, 7, 247, 456)
	Nitric oxide, INF-gamma, and microparticle release	↓	
	TNF-alpha and IL-12 production	↓	
Dendritic cells	Myeloid dendritic cell activity	↓	(7, 247)
	INF-gamma release from plasmacytoid dendritic cells	↑* ↓**	
Antigen presenting cells	MHC II expression	↓	(7)
CD4+ T cells	Th2 pathway differentiation	↑	(6, 247)
	IL-4 production and Treg differentiation from CD4+ T cells	↑	
	INF-gamma release, proliferation, T cell dependent antibody responses, Th1 and Th17 differentiation, IL-6 receptor expression	↓	
CD8+ T cells	INF-gamma production and cytotoxicity	↓	(247)
B cells	Class switch recombination and T cell dependant antibody responses	↓	(247)
	Primary and secondary B cell antibody responses	↑	

*At low concentrations of progesterone

**At high concentrations of progesterone

Supplementary Table 3. Effects of androgens on the immune system

Cell type	Function	Effect	Ref
Thymocyte	Proliferation	↓	(6, 457)
	Apoptosis of CD4+CD8+ double positive thymocytes	↑	
Dendritic cells	MHC II expression	↓	(458)
	Pro-inflammatory cytokine production	↓	
	Anti-inflammatory cytokine production	↑	
	Activation by TLRs	↓	
Neutrophils	Differentiation	↓	(8, 458)
	Production of IL-10 and TGF-beta	↑	
	Production of reactive oxygen species, pro-inflammatory cytokines, and chemokines	↓	
Macrophages	Production of TNF-alpha, IL-1-beta, and chemokines	↓	(8, 458)
	Expression of TLR-4	↓	
	Synthesis of pro-inframammary transcriptional regulators (e.g., NF-kB) and pro-inflammatory products (e.g. TNF-alpha and nitric oxide synthase)	↓	
T cells	Th1 pathway	↓	(7, 8, 457, 458)
	Th2 pathway	↑	
	Apoptosis	↑	
	Proliferation	↓	
	Tregs	↑	
B cells	Immature B cells	↑	(7, 8, 458)
	Immunoglobulin production	↓	

Supplemental Table 4. Hormonal variations during life.

	Observed physiologic changes	Clinical implication	Ref.
Puberty	• Females: skeletal growth, ovulation and the development of menses, breasts, and body hair.<br>• Males: skeletal growth, testicular growth, penis elongation, body hair development, and spermatogenesis.	• From infancy to puberty, there are minimal differences in the skin of males and females.<br>• After puberty, there is apocrine gland maturation and decrease in dermal collagen content.<br>• Females:<br>o More development of subcutaneous fat<br>o The hormonal variations in progesterone and estrogen observed throughout the menstrual cycle lead to multiple skin changes given the important role of these hormones in	(17, 459-461)

		cutaneous homeostasis, as described above.<br>• Males: higher levels of sebum production correlating with male predisposition to acne vulgaris.	
Pregnancy	• Hormonal changes include alterations in estriol, progesterone, prolactin, early pregnancy factor (EPF), alpha fetoprotein (AFP) and leptin levels, which lead to temporary and reversible variations in the immune system of females (as described above).<br>• In addition to sexual organs, there are other immunologic anatomical changes such as a reduction in thymus and bone marrow cellularity.	• Hyperpigmentation<br>• Vascular proliferation<br>• Striae formation<br>• Increased eccrine sweating and decreased apocrine gland activity<br>• Specific pregnancy-related dermatoses can be observed.<br>• Enhanced T regulatory response creates a state of selective maternal tolerance to own antigenically dissimilar fetus, while maintaining peripheral maternal immune competence.<br>• In contrast to most Th1-mediated diseases that improve during pregnancy, Th2 dominant diseases such as atopic dermatitis worsen in the second or third trimester.	(7, 247, 462-469)
Post-partum	• Immunologic recovery state resulting in enhanced Th1 activity and proinflammatory responses<br>• Many anti-inflammatory and immunomodulatory markers that had increased during pregnancy decrease post-partum.<br>• As a result of placental removal post-partum, estrogen, progesterone, estradiol, and prolactin levels (in non-breastfeeding females) decrease abruptly.	• Due to the enhanced Th1 activity that occurs in the post-partum period, many autoimmune disorders that improve during pregnancy flare-up post-partum. For some diseases, this increased risk persists for the first 3 months before returning to pre pregnancy state.	(469-471)
Menopause	• Decreased estrogen, progesterone levels and increased follicle stimulating hormone.	• Skin thinning, decreased number of collagen fibers, elasticity, water content, hydrophilic	(7, 15, 17, 472-474).

	• Post-menopausal females are relatively protected from immunosenescence.<br>• Decreased CD4+ T and B cells<br>• More robust innate inflammatory response with enhanced production of IL-1-beta, IL-6, TNF-alpha, IFN-gamma, and MCP-1 upon antigenic stimulation.	glycosaminoglycans concentrations, bone loss and delayed wound healing.	
Andropause	• Decrease in testicular function, decreased libido, bone loss	• Due to the regulation of sebaceous glands and hair growth by androgens, manifestations of andropause include a decrease in skin thickness and body/scalp hair.	(475, 476)

Supplementary Table 5. Effect of different habits on the integumentary system.

Habit	Effect	Ref.
Cigarette smoking	Nail discoloration, oral mucosa lesions, dyspigmentation, increased risk of skin and mucosal cancers, premature skin aging through the formation of free radicals, altered cellular defense responses, impaired skin repair functions, dysregulation of dermal components, mutagenesis, and exacerbation of multiple dermatoses.	(477)
Alcohol abuse	Jaundice, hyperpigmentation, pruritus, urticaria and vascular changes, and exacerbation of multiple skin conditions such as psoriasis.	(478, 479).
Cannabis	Although cannabis can have an impact on the integumentary system, it is also currently being investigated and used off-label to treat numerous skin diseases.	(480)

Supplementary Table 6. List of skin conditions that may be overrepresented in transgender women and transgender men.

Transgender women	Transgender men
• Xerosis<br>• Hair/nail fragility<br>• Asteatotic dermatitis<br>• Pseudofolliculitis barbae<br>• Melasma<br>• HIV-related dermatoses	• Acne<br>• Androgenic alopecia<br>• HIV-related dermatoses
